# Expression of estrogen receptor beta correlates with adverse prognosis in resected pancreatic adenocarcinoma

**DOI:** 10.1186/s12885-018-4973-6

**Published:** 2018-10-29

**Authors:** Hendrik Seeliger, Ioannis Pozios, Gerald Assmann, Yue Zhao, Mario H. Müller, Thomas Knösel, Martin E. Kreis, Christiane J. Bruns

**Affiliations:** 1 Department of General, Visceral and Vascular Surgery, Campus Benjamin Franklin, Charité Universitätsmedizin Berlin, corporate member of Freie Universität Berlin, Humboldt-Universität zu Berlin, and Berlin Institute of Health, D-12200 Berlin, Germany; 2 Institute of Pathology, Ludwig-Maximilians-University, D-80337 Munich, Germany; 3 Department of General, Visceral and Transplantation Surgery, Hospital of the University of Munich, D-81377 Munich, Germany; 4 Department of General, Visceral and Cancer Surgery, University of Cologne, D-50937 Cologne, Germany; 5 Department of Minimal Invasive and Visceral Surgery, Vivantes Klinikum Neukölln, D-12351 Berlin, Germany

**Keywords:** Pancreatic ductal adenocarcinoma, Pancreatic cancer, Estrogen receptor beta, Prognosis, Survival analysis, Tissue microarray

## Abstract

**Background:**

The relevance of estrogen receptor (ER) expression in pancreatic ductal adenocarcinoma (PDAC) is largely unknown. Clinical trials targeting ER with selective estrogen receptor modulators in pancreatic cancer did not show any benefit. Here, we analyze the impact of recently characterized ER isoform beta on survival in a cohort of patients with resected PDAC.

**Methods:**

Eighty-four patients having undergone pancreatic resection for PDAC at a single institution were identified. Tissue microarrays were constructed of archival tumor specimens. The expression of ER beta was determined by immunohistochemistry and quantified by a system established for estrogen receptor expression in breast cancer. ER beta expression was then correlated with clinicopathological parameters, and univariate and multivariate survival analyses were performed.

**Results:**

Nuclear expression of ER beta was found in 31% of tumors. No significant correlation was found between ER beta expression and TNM status, tumor grade, age or sex. Univariate analysis revealed nodal metastasis and the expression of ER beta as factors correlating with a shorter overall survival and disease free survival. When comparing ER beta expression in patients surviving more than 24 months with those who died from the tumor within 12 or 24 months, respectively, a significantly lower ER beta expression was found in the long term survivors. In multivariate analysis, ER beta expression was demonstrated to be an independent predictor of shorter overall survival.

**Conclusions:**

In resected PDAC, expression of ER beta seems to correlate with poor prognosis. These data may help to identify patients who may benefit from additional systemic therapy including selective estrogen receptor modulators.

## Background

Pancreatic ductal adenocarcinoma (PDAC) is among the leading causes of cancer-related mortality in western countries [1]. In the last decade, overall survival improved only marginally. To date, standard therapeutic regimens consist of surgery, cytotoxic chemotherapy, irradiation, or a combination [2, 3] and result in an overall 5 year survival of less than 10 %. More recently, newer agents targeted against molecular determinants of cancer cells or tumor vessels, or both, have been tested in clinical trials to expand the therapeutic armamentarium [4, 5]. When characterizing molecular targets for potential prognostic and therapeutic use, differences in the sex distribution have led to the investigation of the role of estrogen receptors (ER) in the development and progression of pancreatic cancer and other malignancies [6–11]. Most early trials with the selective estrogen receptor modulator tamoxifen in PDAC yielded only a moderate survival benefit while showing an acceptable safety profile [12–14]. This concept of inhibition of ER mediated effects however did not take into account recently reported existence of differential signaling of ER isoforms ERα and ERβ [15–17].

Human ERβ was cloned in 1996 for the first time and subsequently was shown to have a ligand binding specificity and a signaling response to estrogen agonists that is distinct from ERα [15–18]. While ERα was demonstrated to promote tumor growth and angiogenesis in breast cancer and many other solid tumor types, the role of ERβ is defined much less clearly.

In ERα negative breast cancer specimens, ERβ was shown to correlate with a higher proliferation index [9]. Furthermore, in breast cancer patients, ERβ was characterized as a response marker of the selective estrogen receptor modulator tamoxifen in unselected cohorts and in patients negative for ERα [19, 20]. In non small cell lung cancer patients results are controversial. While in one study, high ERβ expression served as a negative prognostic marker and correlated with a worse outcome [21], a metaanalysis failed to find a consistent correlation of ERβ expression with survival [22]. Beside ERβ-specific effects, ERβ activation seems to interfere with EGF receptor signaling via an activation of the MAP kinase [10, 23].

While pancreatic cancer cell lines were reported to express ERβ [24], there is no consistent information available on the expression of ERβ in human pancreatic ductal adenocarcinoma specimens and its correlation with histopathological parameters and prognostic consequences [8]. The present study was designed to analyze the influence of ERβ expression on overall and disease free survival in PDAC. Here, in a cohort of 94 patients having undergone a pancreatic resection, we correlate ERβ expression in a tissue microarray derived from intraoperative tumor specimens with clinicopathological and survival parameters.

## Methods

### Patients

We identified 111 consecutive patients from a prospective database of patients operated for ductal pancreatic adenocarcinoma at a single institution. Of these, clinicopathological information and prospectively collected follow up data as well as archived tumor material were available for evaluation in 84 patients. Patients with distant metastases were excluded from the study as well as patients who died within 30 days after resection. Specifically, information on age, sex, date of the primary surgery, perioperative irradiation or chemotherapy, TNM tumor status and grading, date last seen, date of death, cause of death, and date of the first identification of tumor progression were extracted from the original patient charts and a regional tumor registry database. The study was approved by the Ethics Committee of the Hospital of the University of Munich. Due to the retrospective nature of the study, explicit consent was not required.

### Tissue microarray construction

Paraffin embedded archive tissue material of tumor and surrounding normal pancreatic tissue was used to generate tissue microarrays (TMAs) after confirming the histological diagnosis of PDAC by a pathologist blinded for the clinical data.

TMAs were prepared essentially as published before [25]. In brief, the area of interest to be sampled was identified and marked on an areal slide corresponding to each paraffin block. Three tissue core biopsies, each 0.6 mm in diameter, were punched out of the donor paraffin block and then arrayed in each of the respective recipient TMA blocks using a manual arrayer (Beecher Instruments, Sun Prairie, WI). Edge confusion was ensured by incubating the TMAs at 37 °C for 30 min. Sections of 2 μm thickness were cut onto adhesive glass slides (Super Frost Plus, Menzel, Braunschweig, Germany).

### Immunohistochemistry

Immunohistochemistry for ERβ was performed using standard technique. Briefly, after deparaffinization and rehydration, slides were blocked with bovine serum albumin. The primary antibody (Rabbit polyclonal to estrogen receptor beta, Abcam, Cambridge, UK), was added in a dilution of 1:200 and incubated overnight at 4 °C. After blocking endogenous peroxidase with 7.5% hydrogen peroxide, a horseradish-peroxidase conjugated polyclonal goat anti-rabbit secondary antibody (Dako, Hamburg, Germany) was added and incubated for 30 min at room temperature. Slides were counterstained with hematoxylin.

### Histopathological evaluation

ERβ expression was quantified analogous to the scoring system proposed by Remmele and Stegner used for ERα in breast cancer [26]. Briefly, staining intensity was scored from 0 (no reaction) to 3 (strong reaction), and the percentage of stained nuclei was scored from 0 (no positive nuclei) to 4 (more than 80% positive nuclei). The scores of staining intensity and stained nuclei were multiplied, yielding a total core of 0 to 12. Positive expression of ERβ was defined as a score of 3 or more. Scoring was performed by two independent pathologists blinded for the clinical data.

### Statistical analysis

Statistical analyses were performed by utilizing IBM SPSS statistics 23 software package (IBM, Armonk, NY). Chi-square tests were applied to test correlation between categorical variables. Survival curves were calculated according to Kaplan-Meier, with differences in survival between strata of low and high ERβ expression and clinicopathological parameters detected by the log-rank test. Multivariate analysis was performed using the Cox proportional hazards model including variables with a *p* value of less than 0.15 in univariate analyses. A *p* value of less than 0.05 was considered statistically significant.

## Results

### Demographic data

The study cohort consisted of 84 patients, 41 men and 43 women with a median age of 65.6 years at the time of the operation (range 32–82 years). Demographic and clinicopathological characteristics of the patients are summarized in Table 1. At the time of the analysis, 63 patients (75.0%) had died from the tumor, and three more patients had tumor progression.

**Table 1:** Clinicopathological parameters of 84 patients with resected pancreatic ductal adenocarcinoma

Variable		*n*	%
Sex	female	43	51.2
	male	41	48.8
Age	≤60 years	24	28.6
	> 60 years	60	71.4
Type of operation	PD	35	41.7
	PPPD	34	40.5
	Distal pancreatectomy	10	11.9
	TP	5	6.0
T status	T1	1	1.2
	T2	10	11.9
	T3	68	81.0
	T4	5	6.0
N status	N0	35	41.7
	N1	49	58.3
Residual tumor^a^	R0	39	48.1
	R1	42	51.9
Histological grading	G1	2	2.4
	G2	28	33.3
	G3	54	64.3
Perioperative therapy	Chemotherapy	5	6.0
	Chemoradiation	49	58.3
	none	30	35.7

*PD* partial pancreatoduodenectomy (Kausch-Whipple procedure), *PPPD* pylorus preserving partial pancreatoduodenectomy (Traverso-Longmire procedure), *DP* distal pancreatectomy, *TP* total pancreatectomy

^a^missing information on resection status in three patients

### Expression of ERβ

**Fig. 1:**
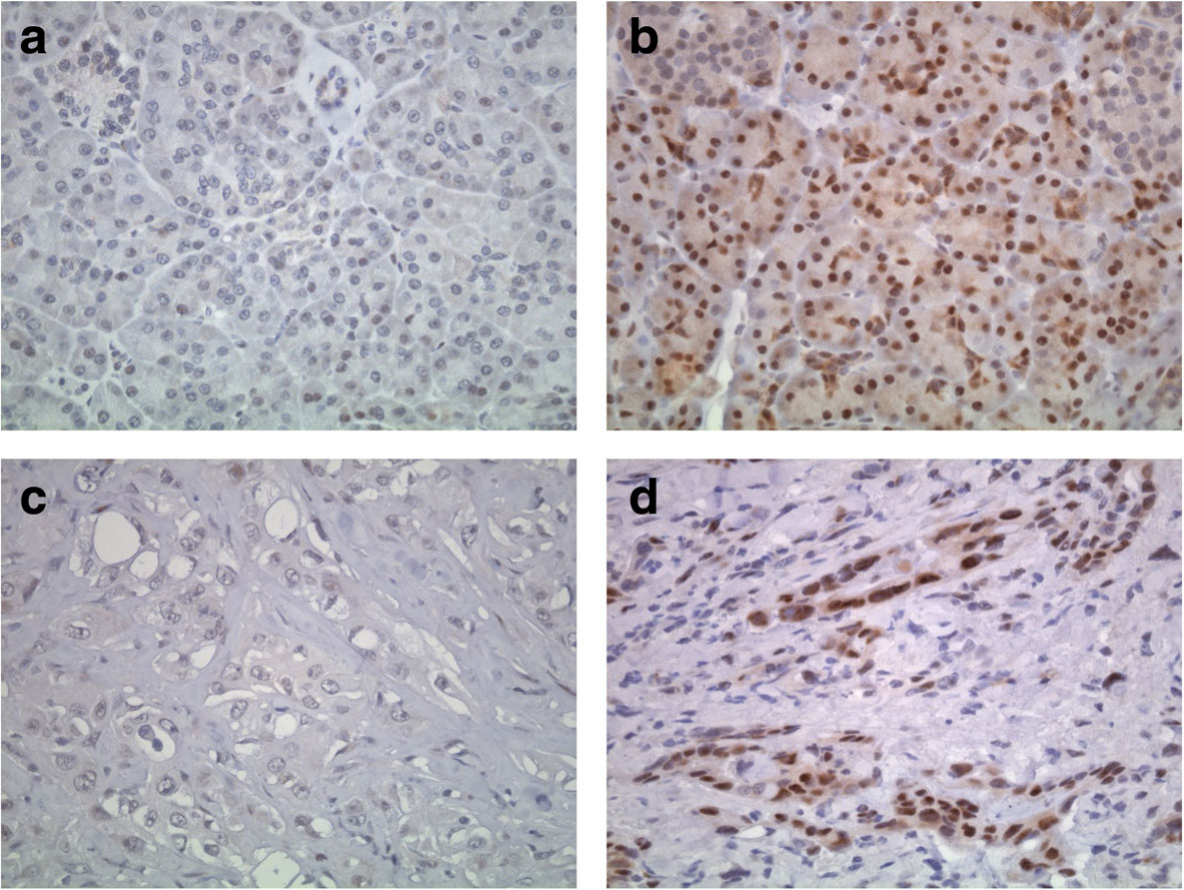
Nuclear expression of estrogen receptor beta (ERβ) in pancreatic ductal adenocarcinoma and corresponding normal tissue. Samples of nontumorous pancreatic tissue (upper panel) and corresponding pancreatic ductal adenocarcinoma (lower panel) without (**a** and **c**) and with ERβ expression (**b** and **d**) are shown. ERβ immunohistochemistry, magnification 640× (**a-d**)

A nuclear expression of the ERβ was detected in 26 PDAC tumor specimens (31.0%). Representative slides are shown in Fig. 1. No correlation was seen between ER expression and other clinicopathological parameters, such as sex, age, T and N stage, and histological grading. Furthermore, additional therapy (chemotherapy or chemoradiation) and ER expression did not correlate (Table 2). Interestingly, in adjacent normal pancreatic tissue, ER beta expression was detected in 41 patients (48.8%). A downregulation of ER beta expression in tumor tissue, compared to normal tissue, as defined by a lower staining score, was seen in 42 cases (50.0%).

**Table 2:** Correlation of estrogen receptor beta (ERβ) expression with clinicopathological parameters

Variable		*n*	ERβ expression	*P* value
Total		84	26 (31.0%)	
Sex	female	43	16 (37.2%)	0.243
	male	41	10 (24.4%)	
Age	≤60 years	24	7 (29.2%)	1.000
	> 60 years	60	19 (31.7%)	
T status	1–2	11	3 (27.3%)	1.000
	3–4	73	23 (31.5%)	
N status	0	35	11 (31.4%)	1.000
	1	49	15 (30.6%)	
Tumor grade	1–2	30	7 (23.3%)	0.328
	3	54	19 (35.2%)	
Residual tumor^a^	R0	39	11 (28.2%)	0.487
	R1	42	15 (35.7%)	
Perioperative therapy	Surgery alone	30	13 (43.3%)	0.086
	Chemoradiation/Chemotherapy	54	13 (24.1%)	

^a^missing information on resection status in three patients

### Univariate survival analysis

**Fig. 2:**
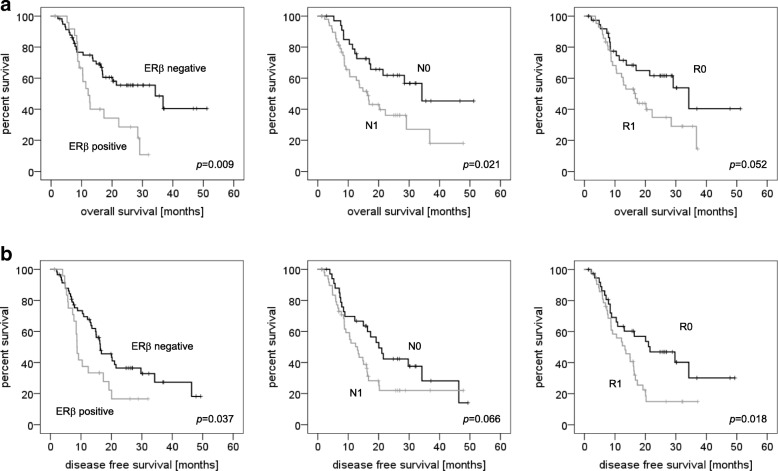
Analysis of overall survival and disease free survival in 84 patients with resected pancreatic ductal adenocarcinoma

**Fig. 3:**
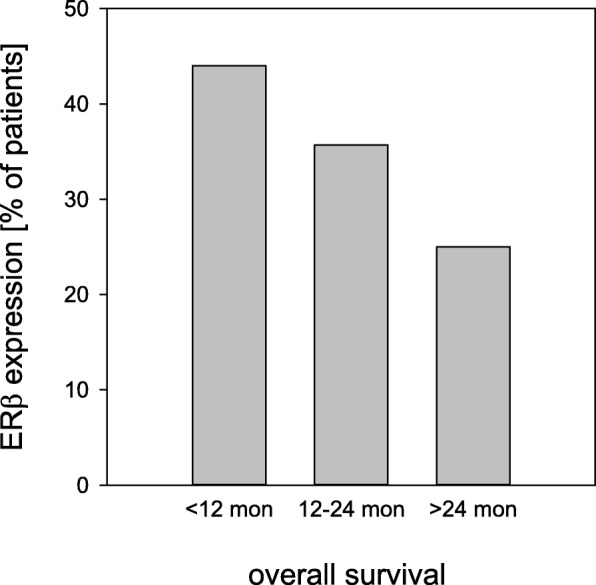
Estrogen receptor beta (ERβ) expression in ductal pancreatic adenocarcinoma. Percentages of ERβ expressing tumors are shown by stratification into tumor dependent death after less than 12 and more than 12 but less than 24 months, and overall survival more than 24 months. Significantly fewer tumors of long-term overall survivors expressed ERβ, compared to other strata (*p* = 0.043, < 24 months versus 12–24 months; *p* = 0.0026, > 24 months versus < 12 months; *p* = 0.000099, > 24 months versus < 24 months)

Mean overall survival of all patients after resection of the primary tumor was 27.0 months (95% confidence interval 22.3–31.6 months), and mean disease free survival was 21.2 months (95% confidence interval 17.2–25.1 months). Patients with ERβ expressing tumors survived 16.6 months compared to 30.9 months in patients without ERβ expression (*p* = 0.009, Fig. 2a). Disease free survival was 13.5 months in patients with ERβ expression compared to 23.5 months in patients with no ERβ expression (*p* = 0.037, Fig. 2b). Overall survival in nodal positive patients was 21.5 months versus 33.1 months in nodal negative patients (*p* = 0.021, Fig. 2a). Disease free survival was 18.5 months in nodal positive patients which was significantly shorter compared to 25.1 months in patients with negative nodal status (*p* = 0.066, Fig. 2b). Details are given in Table 3 and Table 4. In long term survivors (overall survival 24 months and more, *n* = 25) ERβ expression was detected in 25%, while patients who survived less than 12 months (n = 25) showed ERβ expression in 44% (Fig. 3, *p* = 0.0027).

**Table 3:** Univariate analysis of prognostic factors for overall survival in resected pancreatic ductal adenocarcinoma

Variable		Mean OS [months]	95% CI			Median OS [months]	*p* value
Total		27.0	22.3	–	31.6	21.4	
Sex	female	23.1	17.7	–	28.5	16.3	0.122
	male	30.2	22.5	–	37.9	34.2	
Age	≤60 years	29.4	21.6	–	37.1	21.4	0.398
	> 60 years	25.4	20.1	–	30.8	20.2	
T status	T1–2	26.2	15.2	–	37.2	17.0	0.969
	T3–4	26.8	21.8	–	31.9	22.2	
N status	N0	33.1	26.1	–	40.2	34.2	0.021
	N1	21.5	16.1	–	26.8	16.3	
Tumor grading	G1–2	28.1	21.3	–	34.9	36.8	0.228
	G3	25.3	19.6	–	31.0	16.7	
Residual tumor	R0	31.4	24.0	–	38.8	34.2	0.052
	R1	19.8	15.7	–	23.9	16.7	
ERβ expression	negative	30.9	25.2	–	36.7	34.2	0.009
	positive	16.6	12.7	–	20.4	12.2	
Perioperative	CRT/CTX	25.5	18.5		32.5	17.4	0.800
therapy	none	27.2	21.5		32.9	21.4	

*CI* confidence interval, *OS* overall survival, *CRT* chemoradiotherapy, *CTX* chemotherapy

**Table 4:** Univariate analysis of prognostic factors for disease free survival in resected pancreatic ductal adenocarcinoma

Variable		Mean DFS [months]	95% CI			Median DFS [months]	*p* value
Total		21.2	17.2	–	25.1	15.0	
Sex	female	20.4	15.2	–	25.6	12.2	0.485
	male	22.0	16.1	–	27.9	16.3	
Age	≤60 years	22.4	15.0	–	29.8	16.1	0.704
	> 60 years	20.5	15.8	–	25.2	15.0	
T status	T1–2	25.6	13.5	–	37.7	16.3	0.701
	T3–4	20.7	16.4	–	25.0	15.0	
N status	N0	25.1	18.9	–	31.2	20.0	0.066
	N1	18.5	13.6	–	23.5	13.0	
Tumor grading	G1–2	24.4	17.3	–	31.6	20.1	0.293
	G3	19.5	14.8	–	24.1	14.8	
Residual tumor	R0	25.9	19.4	–	32.5	21.5	0.018
	R1	15.1	11.7	–	18.5	13.3	
ERβ expression	negative	23.5	18.7	–	28.4	16.3	0.037
	positive	13.5	9.6	–	17.4	8.7	
Perioperative	CRT/CTX	21.4	16.5	–	26.3	15.0	0.800
therapy	none	20.4	13.5	–	27.2	16.7	

*CI* confidence interval, *DFS* disease free survival, *CRT* chemoradiotherapy, *CTX* chemotherapy

### Multivariate survival analysis

To validate ERβ expression as an independent prognostic indicator in PDAC on overall survival, multivariate regression analysis was performed. Expression of ERβ was demonstrated to be an independent prognostic indicator of overall survival (hazard ratio 1.938, *p* = 0.047). Of the remaining variables tested only positive nodal status showed a trend towards adverse survival however did not become statistically significant (hazard ratio 1.831, *p* = 0.069). Male sex and residual tumor status also failed to show statistical significance in the multivariate survival analysis. Details are shown in Table 5.

**Table 5:** Multivariate analysis of prognostic factors for overall survival in pancreatic ductal adenocarcinoma

Parameter	Hazard ratio	95% CI	*p* value
ERβ expression	1.938	1.010–3.720	0.047
N1	1.831	0.954–3.517	0.069
Residual tumor	1.704	0.894–3.247	0.105
Male sex	0.628	0.355–1.311	0.251

*CI* confidence interval

## Discussion

In the present study, ERβ was expressed on PDAC in 31% of all patients. Expression of ERβ did not correlate with any of the clinicopathological parameters examined, however ERβ expression was strongly associated with an adverse overall survival and disease free survival in univariate analyses. Multivariate analysis showed that ERβ expression on tumor cells was an independent prognostic factors of overall survival.

To our knowledge, this study is the largest series on expression of ER on pancreatic neoplasms. The fact that ERα is not detectable with immunohistochemical methods on PDAC tissue is concordant with several other smaller studies [27, 28]. However, there are two studies which showed ERα expression on mRNA level on PDAC [29, 30]. Whether this finding reflects ERα protein levels being expressed in very small amounts not detectable with immunohistochemical methods, or a missing translation of ERα mRNA in PDAC is unknown. Interestingly, mucinous cystic tumors seem to express ERα more frequently than PDAC, possibly reflecting the “ovarian-type stroma” defining mucinous cystic tumors [31, 32]. Data on ERα expression in this entity is still pending.

ERβ was however expressed in nontumorous tissue, and to a lesser extent in the corresponding PDAC specimens. Compared to normal pancreatic tissue, an overall loss of ERβ expression in PDAC was detected in the majority of the investigated cases, suggesting ERβ loss as a molecular event in the line of tumor progression. Yet, the presence of ERβ expression in the tumor correlates with an adverse prognosis. This phenomenon may be explained by a crosstalk of ERβ signal transduction and other pathways that are activated during of tumor progression, leading to a more aggressive tumor phenotype in those subjects with an unchanged ERβ signaling pathway. In fact, non-ligand dependent ER signaling is well characterized. The ligand dependent pathway of ER signaling is initiated by steroid ligand binding to the ER. In the non-ligand dependent pathway, activated kinase growth factor receptors phosphorylate ER, leading to its activation [33–36]. Loss of ERβ expression during tumor progression was described in several other tumor entities [6, 37, 38]. Seemingly, interplay between ERβ expression and tissue specific distribution of growth factors may be important for subsequent tumor progression.

In colon cancer cell lines, ERβ was shown to be the predominant ER, whereas ERβ mRNA expression was a lot lower and similar to normal tissue [6]. Similar results were obtained when examining ERβ expression in human tumor samples [37, 39].

No significant correlation was found between ERβ expression and clinicopathological features of patients and PDAC specimens. A trend was seen towards a lower ERβ expression in male patients, but statistical significance was not reached. Circulating estrogen levels may have some effect on ERβ expression. In fact, an upregulation of ERβ by estrogen has been described previously [40]. However, since the majority of the female patients in our cohort is postmenopausal, as reflected by age distribution, this effect must be regarded as questionable. Similarly, a trend was detected towards a higher expression of ERβ in less differentiated tumors, underlining a role of ERβ in tumor progression towards a more aggressive phenotype. This finding is supported by recent data in breast cancer, where ERβ expression was found to correlate with tumor grading and higher expression of the proliferation marker Ki-67 in women with ERα negative breast cancer [9]. Similarly, in esophageal cancer, a correlation of ERβ expression with poor differentiation status and tumor stage was found in squamous cell carcinomas and in adenocarcinomas [41].

In the analyzed cohort, ERβ expression was found to strongly correlate with a reduction of overall survival and disease free survival in patients with resected pancreatic adenocarcinoma in univariate and multivariate analyses. Multivariate analysis of overall survival, revealed that the expression of ERβ is an independent negative prognostic factor. Patients with lymph node metastases had a shorter survival, although this did not reach statistical significance. Interestingly, resection status (R0 versus R1) also failed to be a statistically significant prognostic factor of overall survival and disease free survival in the multivariate analysis. This finding may be partially explained by underestimation of the number of R1 resections [42], as examination of circumferential resection margin was not incorporated in routine pathological protocol when specimens were analyzed originally.

At present, clinical data on the impact of ER expression on solid tumors on survival except in breast cancer is not sufficient to establish a clear prognostic role of the different ER subtypes. In esophageal squamous cell carcinoma and gastric adenocarcinoma, expression of ERα in the absence of ERβ was described to correlate with an adverse prognosis [38, 43]. In colorectal cancer, loss of ERβ expression correlates with advanced cancer stages and poor survival [44]. In hepatocellular cancer, both ERα and ERβ are expressed [45]. Presence of a variant ERα in hepatocellular carcinoma correlates with shorter survival, compared to wild type ER [46].

However, some clinical and experimental data support the hypothesis that ERβ expression may lead to a more aggressive tumor phenotype. In ERα negative breast cancer, ERβ expression correlates with an increased Ki-67 expression, suggesting a higher proliferation rate within the tumor cells. In the same cohort, ERβ expression positively correlated with advanced tumor grade [9]. Similar findings were described in sarcoma patients [11]. In vitro proliferation of non small cell lung cancer cells was reduced by siRNA mediated elimination of ERβ signaling [10]. In contrast, ERβ deficiency results in an enhanced tumorigenesis in the small bowel, but not in the colon of Apc (min/+) mice, suggesting a tumor suppressor effect of ERβ [47].

The exact role of ER signaling in solid tumors remains to be defined. Our data strongly suggest a tumor promoting role of ERβ signaling in PDAC, which is in line with previously published data on non small cell lung cancer [21, 48]. However, there are studies showing an effect of ERβ on tumor suppression, especially in colon cancer [49–51]. These conflicting results may be explained by differences in the tissue distribution of the ER subtypes and their splicing variants. Moreover, ER signaling is embedded in a complex signaling network controlling tumor cell growth and proliferation to the effect that context specific signaling interactions lead to different effects in different tissue types [52]. In the present study, the phosphorylation status of ERβ was not examined. One can speculate that differences of tissue specific ERβ phosphorylation lead to differential ER mediated actions that are mediated by ligand independent ER signaling. Specifically, an extensive crosstalk between epidermal growth factor receptor (EGFR) and ER mediated pathways is well documented in several tumor types [40]. Since a strong EGFR expression is present in PDAC, ER phosphorylation by EGFR mediated growth signals and the resulting proliferative stimulus may be an important contributor to the adverse prognostic effects observed. In fact, EGFR inhibition combined with ER signaling disruption resulted in a marked inhibition of tumor xenograft growth [10].

A limitation of the present study is the different perioperative treatment of the patients included. In the cohort presented, 58% of the patients received perioperative chemoradiation. Although a clear survival benefit of perioperative chemoradiation in patients with resectable PDAC has not been shown [53], there is a possible impact of irradiation and/or chemotherapy on ER expression [54]. However, whether this is true for the ERβ subtype and is unknown, and its clinical significance remains unclear. To clarify this issue, additional studies may correlate the expression of ERβ on pretherapeutic tumor biopsies with the expression on surgical specimens after neoadjuvant therapy.

## Conclusions

Here, the expression of ERβ was analyzed on surgical specimens of patients with PDAC and correlated with overall and disease free survival. ERβ was expressed on 31% of PDAC surgical specimens. A correlation between ERβ expression and an adverse prognosis in resected PDAC seems to exist. These data may be useful in defining a role of ERβ expression as a prognostic indicator and as a potential molecular target in patients with advanced PDAC.

## Abbreviations

CI: Confidence interval
CRT: Chemoradiotherapy
CTX: Chemotherapy
DFS: Disease free survival
DP: Distal pancreatectomy
EGF: Epidermal growth factor
EGFR: Epidermal growth factor receptor
ER: Estrogen receptor
MAP kinase: Mitogen activated protein kinase
OS: Overall survival
PD: Partial pancreatoduodenectomy
PDAC: Pancreatic ductal adenocarcinoma
PPPD: pylorus preserving partial pancreatoduodenectomy
TMA: Tissue microarray
TP: Total pancreatectomy
